# Adaptive Dynamics Simulation of Interference Phenomenon for Physical and Biological Systems

**DOI:** 10.3390/e25111487

**Published:** 2023-10-26

**Authors:** Tadashi Ando, Masanari Asano, Andrei Khrennikov, Takashi Matsuoka, Ichiro Yamato

**Affiliations:** 1 Department of Applied Electronics, Tokyo University of Science, 6-3-1 Niijuku, Katsushika-ku, Tokyo 125-8585, Japan; tando@rs.tus.ac.jp; 2 Department of Information and Computer Science, Faculty of Humanity-Oriented Science and Engineering, Kindai University, 11-6 Kayanomori, Iizuka-shi, Fukuoka 820-8555, Japan; asano@fuk.kindai.ac.jp; 3 International Center for Mathematical Modelling in Physics and Cognitive Sciences, Linnaeus University, SE-351 95 Växjö, Sweden; 4 School of General Education and Management Studies, Suwa University of Science, 5000-1 Toyohira, Chino, Nagano 391-0292, Japan; matsuoka@rs.sus.ac.jp; 5 Department of Biological Science and Technology, Tokyo University of Science, 6-3-1 Niijuku, Katsushika-ku, Tokyo 125-8585, Japan; iyamato@rs.noda.tus.ac.jp

**Keywords:** two-slit interference, adaptive dynamics, interaction network, quantum mechanics, particle model simulation

## Abstract

Biological systems have been shown to have quantum-like behaviors by applying the adaptive dynamics view on their interaction networks. In particular, in the process of lactose–glucose metabolism, cells generate probabilistic interference patterns similarly to photons in the two-slit experiment. Such quantum-like interference patterns can be found in biological data, on all scales, from proteins to cognitive, ecological, and social systems. The adaptive dynamics approach covers both biological and physical phenomena, including the ones which are typically associated with quantum physics. We guess that the adaptive dynamics can be used for the clarification of quantum foundations, and the present paper is the first step in this direction. We suggest the use of an algorithm for the numerical simulation of the behavior of a billiard ball-like particle passing through two slits by explicitly considering the influence of the two-slit environment (experimental context). Our simulation successfully mimics the interference pattern obtained experimentally in quantum physics. The interference of photons or electrons by two slits is known as a typical quantum mechanical effect. We do not claim that the adaptive dynamics can reproduce the whole body of quantum mechanics, but we hope that this numerical simulation example will stimulate further extensive studies in this direction—the representation of quantum physical phenomena in an adaptive dynamical framework.

## 1. Introduction

Recently, we formulated a quantum-like (QL) algorithm that is generally applicable to various phenomena of living systems from the viewpoint of adaptive dynamics [1,2]. They show interference phenomena as the violation of classical total probability law (TPL), such as adaptation, evolution, and cognition. These interference phenomena have been envisaged as the effects of the entanglement of the interactions between the environment and composite members. Further, we have shown that such formulation can also be applicable to phenomena other than living systems with complex interacting networks, such as the simplest physical three-body system [3]. Thus, it is probable that any systems with more than two interactions at a time on at least one component could be universally formulated by adaptive dynamics by considering every interaction in the environment.

A double-slit system of photons or electrons also generates the interference pattern, i.e., a two-slit pattern is not a mere summation of two one-slit patterns, which is known as a typical quantum mechanical effect. Thus, it would be interesting to examine whether the adaptive dynamical approach can reproduce a similar interference pattern.

In a series of papers [1,2,3], we realized the following research pathway: We started with an application of the standard mathematical formalism of quantum mechanics outside of physics—so-called quantum-like modeling—to model theoretically non-classical statistical data, which can be found on all biological scales, from proteins and cells to cognitive, ecological, and social systems. In [1,2,3], we called this field of research quantum bioinformatics. The latter should not be mixed with quantum biophysics, which is the field devoted to studying genuine quantum physical processes in biosystems. According to Bohr [4], the quantum theory provides a probabilistic description and prediction for the outcomes of observations over quantum physical systems. The question of whether a finer description of micro-phenomena is possible is the basic question of quantum foundations (see, e.g., the article by Einstein–Podolsky–Rosen (EPR) [5]). In addition to using various proposals for the interpretation such as the probabilistic description [4] and path integral by Feynman [6], attempts to find a proper answer to it lead to intensive theoretical and experimental studies within various hidden variable theories culminating in the Nobel Prize in Physics (2022) for the experimental confirmation of the violation of the Bell inequalities for spatially separated systems. In quantum physics, the no-go output for hidden variable theories is commonly accepted, and it matches the Bohr statement on the completeness of quantum mechanics. In contrast, in quantum-like modeling for biological systems, one is not satisfied by obtaining the tool for operation with experimental probabilistic data—the mathematical formalism of quantum theory. In biology, the quantum operational description cannot be considered as the final and finest possible one. A more detailed description of the biological phenomena matching with quantum-like statistical behavior is demanded. The studies in this direction were started by Ohya et al., and they lead to the theory of the adaptive dynamical description of quantum-like phenomena, including the interference of probabilities and the Bell inequalities [1]. Adaptive dynamics is context-sensitive, and it is about contextually hidden variable models in the common language of the hidden variables theory. The adaptive dynamical simulations of the interference effect are important for the quantum interaction project (quantum-like modeling in biology, decision making, psychology, cognition, etc.) [7,8,9,10,11,12,13,14]. Success (at least partial) in the adaptive dynamical modeling of quantum-like phenomena in biology stimulated us to guess that it might be that the adaptive dynamics can be used as a tool for the contextual modeling of genuine quantum physical phenomena. So, after a long journey to biology, in this paper, we are back to physics and we intend to apply the experience of quantum-like modeling.

In addition to our adaptive dynamical view in biology [2], there have been plenty of papers reporting the analogy of biological and macroscopic phenomena with quantum mechanics (see review [15]). The author of this review discussed such analogical behavior in not only biology but also in many complex systems such as bifurcations and self-organization, revealing symmetry breaking as the common basic property. He further speculated that symmetry breaking may occur due to the quantum fluctuations of the vacuum. In adaptive dynamics, we attributed the origin of the quantum-like behavior to interactions with the environment [1,2,3]: Everything is interconnected. This concept enabled us to perform probabilistic modeling and a simulation on the basis of classical dynamics or the probability theory [3].

There are many papers devoted to the classical probabilistic modeling of quantum phenomena, starting with an article [16]. One of the authors published a monograph on this topic [17]. Specifically, our study is a part of the research on the numerical simulation of quantum and quantum-like phenomena within the classical mechanical framework (for example, [18,19,20,21,22]). Among them, Pascasio et al. performed a classical simulation of a two-slit situation [23,24,25]. But none of them incorporated the interaction effect from the environment. In this sense, our adaptive dynamics approach to two-slit interference is the first challenging trial.

**Figure 1:**
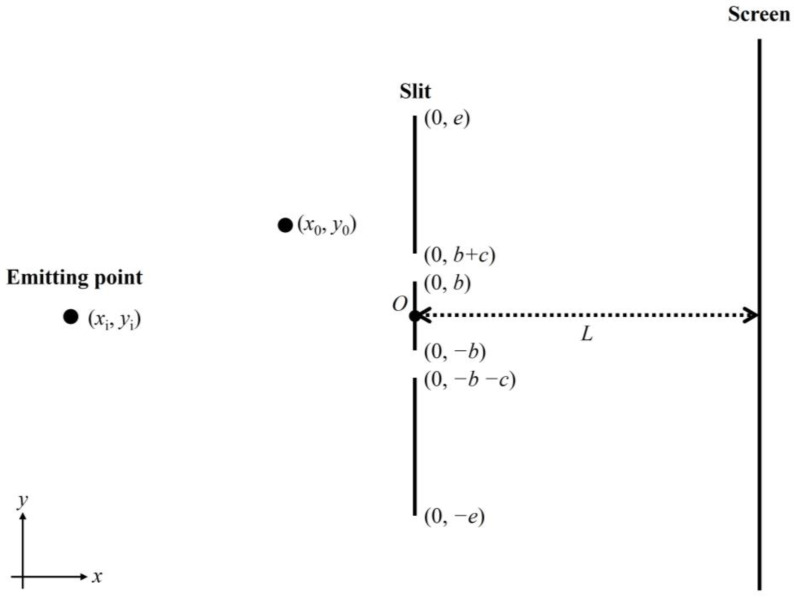
Description of the simulation system. As described in Section 2, *x*-axis shows the emitted direction of a particle. At *x* = 0, a slit wall is placed perpendicular to the *x*-axis, showing the *y*-axis. The slit spacing, width, and wall length are “2*b*”, “*c*”, and “2*e*”, respectively. The distance-dependent part of the potential exerted by the slit system with the particle position (*x*_0_, *y*_0_) is *U*(*x*_0_, *y*_0_) and is shown in Section 2.

In this paper, we considered the two-slit system under the influence of the total environmental interaction. We simulated a billiard ball-like particle passing through the two slits; the simulation condition is shown in Figure 1. The obtained pattern mimicked well the interference phenomenon or the wave-like/quantum-like property. Based on our successful simulation, we discuss the possibility of adaptive dynamics as a candidate of the principle of quantum mechanics by extending the concept obtained in the biological and macroscopic world: “everything in this world is under the interaction network with its environment producing the entanglement and quantum-like behavior”.

## 2. Methods

Assumptions of the interaction: The interaction is assumed to be between the materials composing the slit wall and the permeating particle. In other words, we thought that the particle changes its momentum by interacting with the potential created by the slit wall. So far, the scientific data about such interaction of the particle with its environment are not well known; we just assumed appropriate artificial potential and random walk behavior. In this study, we assumed that the interaction has the potential to be equivalent to the repulsive interaction obeying the inverse square law, and its strength was adjusted.

Simulation condition: The motion of a particle like a billiard ball is simulated according to the collision dynamics [26] interacting with the slit wall potential and the random walk perturbation at every time step “*dt*”. The number of emitted particles is “*a*” (generally 2,000,000). The position of the particle is (*x*_0_, *y*_0_), the emitted direction is along the *x*-axis, and the wall with the slits is placed vertically in the *y*-axis direction at *x* = 0 (Figure 1). Point particle emission (*y_i_* = 0) or parallel particle emission at (*x_i_*, *y_i_*) is used; the slit spacing is “2*b*”; the slit width is “*c*”; the particle initial velocity (*v_xi_*, *v_yi_*) is (1, 0); the screen is placed at *x* = *L*; the length of the slit wall is “2*e*”; and the particle interacts with the slit wall at every time-step “*dt*” with the strength of “*ratio*”. In the case of parallel particle emission, the *y_i_* position of the emission position is randomly moved between *y_i_* = 0 ± (2*b* + *c*). The degree of random motion of the particle is “*con*”. “*dt*”, “*ratio*”, and “*con*” are adjusted depending on the slit conditions used to obtain successful patterns. Simulations were performed on Intel Xeon 5160 Dual CPU.

Effect of the interaction with slit wall and random motion on particle velocity: First, the distance-dependent part of the potential *U*(*x*_0_, *y*_0_) can be obtained as the following formula, and the force (*F*_x_, *F_y_*) is obtained as its gradient (see Figure 1):

(1) $$U\left(x_{0},y_{0}\right)=\int_{-e}^{e}\frac{1}{\sqrt{x_{0}^{2}+{\left(y-y_{0}\right)}^{2}}}dy-\int_{b}^{b+c}\frac{1}{\sqrt{x_{0}^{2}+{\left(y-y_{0}\right)}^{2}}}dy-\int_{-b-c}^{-b}\frac{1}{\sqrt{x_{0}^{2}+{\left(y-y_{0}\right)}^{2}}}dy$$

The *x*- and *y*-direction velocities (*v_x_*, *v_y_*) of the particle are assumed to change by adding “*ratio*” × *F_x_* and “*ratio*” × *F_y_*, respectively, depending on the degree of interaction strength “ratio”. In addition, in the case of random movement, the velocity changes in “*con*” × (uniform random number between ±0.5) are added. Moreover, the absolute value of the velocity changed by these interactions and random motion is renormalized to the initial velocity each time. The pseudo code for this part is as follows:

$$v_{x}=v_{x}+ratio\cdot F_{x}+con\cdot \left(rand-0.5\right)$$

$$v_{y}=v_{y}+ratio\cdot F_{y}+con\cdot \left(rand-0.5\right)$$

$$v_{x}=v_{0}\cdot v_{x}/\sqrt{v_{x}^{2}+v_{y}^{2}}\;$$

$$v_{y}=v_{0}{\cdot v}_{y}/\sqrt{v_{x}^{2}+v_{y}^{2}}\;$$

$$x_{0}=x_{0}+v_{x}\cdot dt$$

$$y_{0}=y_{0}+v_{y}\cdot dt$$

Here, *rand* is the uniform random number between 0 and 1, and $v_{0}=\sqrt{v_{xi}^{2}+v_{yi}^{2}}$.

## 3. Results

### 3.1. In Case of Parallel Emission of a Particle

**Figure 2:**
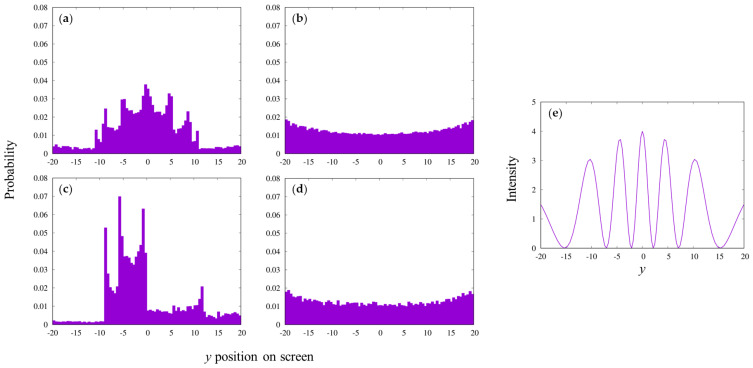
Simulated interference pattern. (**a**) The simulated result is shown with the following condition: slit width, 0.1 (*c*); slit spacing, 0.6 (2*b*); length of the slit wall, 2000 (2*e*); initial velocity, 1 (*v_xi_*) in *x* direction; time step, 0.3 (*dt*); emission position, *x_i_* = −12 (−40 × *dt*); interaction strength, 0.14498 (*ratio*); screen position, 100 (*L*); number of emitted particles, 2,000,000. On screen, 80 samples were counted with the sampling bin width of 0.5. The number of the arrived particles at each position on the screen divided by the total number arrived (18,442) was plotted as the probability. (**b**) The 1-slit pattern with the total number emitted (4,000,000) and arrived (141,258). (**c**) The pattern obtained by counting particles passing through only one of the 2 slits; total number arrived (9095). (**d**) The pattern obtained with the different emission position, *x_i_* = −11.985, from the condition in (**a**). Total number arrived was 22,839. (**e**) Fraunhofer diffraction pattern for a double slit. The pattern is described by the following equation [27]: $\frac{I\left(\theta \right)}{I\left(0\right)}=4{\left\{\frac{\sin\left[\left(\frac{\pi b}{\lambda }\right)\sin\theta \right]}{\left(\frac{\pi b}{\lambda }\right)\sin\theta }\right\}}^{2}\cos^{2}\left[\left(\frac{\pi a}{\lambda }\right)\sin\theta \right]$, where *I* is the intensity, θ is the scattering angle from the center of the slit, λ is the wavelength, a is the separation, and b is the slit width. For this plot, a=0.7, b=0.1, and λ=0.2 were used. The *y* position was calculated via y=15tan⁡θ.

Even without random walk, the interference that is typical to the experimental observation was reproduced by allowing the particles to pass through the slits under the interaction with the wall (Figure 2).

Figure 2a shows three peaks on both sides. Additionally, the light wave interference was theoretically calculated under a similar condition (Figure 2e), corresponding to that in Figure 2a. The patterns resemble each other, although not quantitatively.

A single slit produced a uniform/slightly concave distribution (Figure 2b). The concave shape is unique for our simulation, and it is different from the experiments. We did not examine further the difference in the detail. Interestingly, it shows a small wave-like pattern on both sides, which may correspond to a kind of Fraunhofer diffraction [27]. When the particles through one of the two slits were not counted with the two-slit environment that was kept, a partial interference pattern was observed (Figure 2c), and the addition of two such patterns resulted in the original two-slit pattern; this is apparently different from the results of the “which path experiments” (such as in ref. [28]). When the particle emission position was slightly shifted (*x_i_* = 12 − 0.015), the pattern changed dramatically, and no interference was obtained (Figure 2d). This indicates that the positional relationship between the incident particle and the slits is important, which is not observed in the case of the optical two-slit experiments. This is a part that does not correspond to the actual experimental results, which may be due to our simulation model being too simple.

**Figure 3:**
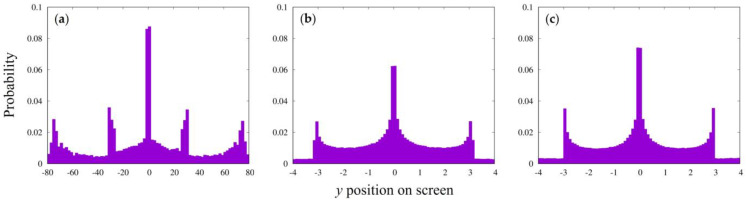
Interference pattern for other conditions with parallel emission particles. (**a**) The case of slit width of 1, slit spacing of 6, time step of 3, interaction strength of 1.385, particle emission position of −120, screen position of 220, 80 samplings with bin width of 2, and total number of particles emitted and arrived at the screen of 2,000,000 and 53,810, respectively. (**b**) The case of the slit width of 0.04, slit spacing of 0.06, time step of 0.04, interaction strength of 0.0208, particle emission position of −4, screen position of 24, 80 samples with sampling bin width of 0.1, and total number of particles arrived at the screen of 556,605. (**c**) The case of slit width of 0.04, slit spacing of 0.06, time step of 0.03, interaction strength of 0.0171, particle emission position of −3, screen position of 48, 80 samples with sampling bin width of 0.1, and total number of particles arrived at the screen of 529,131.

Similar interference patterns were obtained under other slit conditions (Figure 3). The two patterns, shown in Figure 3b,c, may represent the corresponding set of using two different wavelengths of light for the same slit condition.

### 3.2. In the Case of Point Emission of a Particle

Random movement was essential in the simulation for the case of point particle emission. The random motion was assumed to be derived by receiving the similar influence as the interaction with the slit wall from all other environments including the universe. As a result, such random motion is expected to produce a particle flux near the slit similar to the parallel particle beam described above.

**Figure 4:**
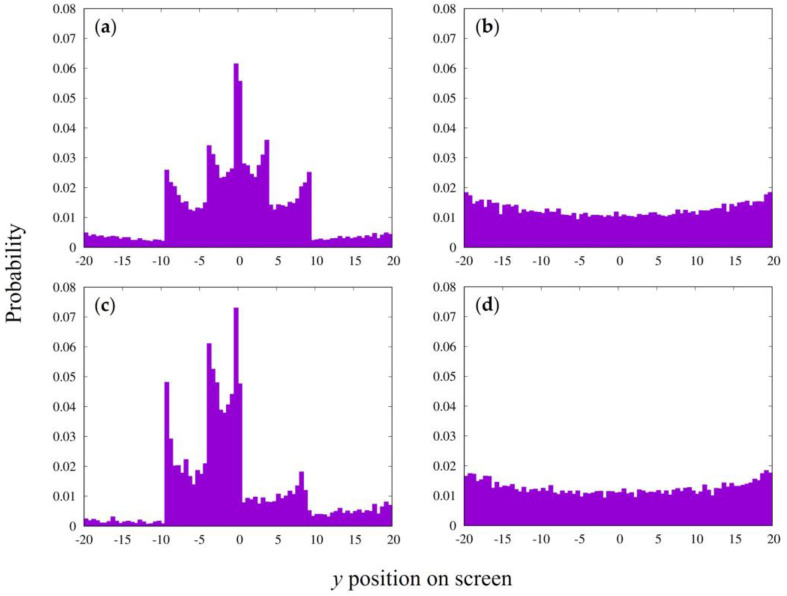
Interference pattern obtained with point emission particles. (**a**) The slit conditions are the same as those in Figure 2a (slit width of 0.1; slit spacing of 0.6), with the point particle emission position at *x_i_* = −9000 and the degree of random motion *con* = 10^−6^. Total numbers of emitted and arrived particles were 2,000,000 and 15,980, respectively. (**b**) One-slit pattern with the same condition as that in (**a**). Total numbers of emitted and arrived particles were 4,000,000 and 25,634, respectively. (**c**) The pattern obtained by counting arrived particles passing through only one of the 2 slits, corresponding to that in (**a**). Total number of arrived particles was 7962. (**d**) Interference pattern with the point particle emission position at *x_i_* = −8999.985. Other conditions were the same as those for (**a**). Total number of arrived particles was 19,134.

Figure 4 shows the simulation pattern obtained with a slit width of 0.1 and a slit spacing of 0.6, same to that in Figure 2a. The pattern (Figure 4a) is typical as that experimentally obtained for the two-slit situation. The third outermost peaks observed in Figure 2a seem to be missing, but we did not investigate it further. A similar pattern was reproduced even by changing the initial emission position from −6000 to −60,000 as long as the distance was the integer multiple of “*dt*”. A single slit produced a similar uniform/slightly concave distribution (Figure 4b) as that in Figure 2b. When the particles through one of the two slits were not counted or the emission position was slightly shifted (*x_i_* = 18,000 − 0.015), the obtained patterns were similar (Figure 4c,d) to the case of the parallel emission simulation shown in Figure 2.

It was not possible to reproduce the interference when only random motion was taken into account; an interaction with the two-slit wall was essential. Our limited simulation result may not exclude the proposal by Nelson [29] that the stochastic process alone produces the interference, but we would rather doubt how the particle recognizes the existence of the two pathways and how its stochastic process catches the entangled environment of the two-slit situation. An interaction with the two-slit wall should be essential. This is the main question in this study.

**Figure 5:**
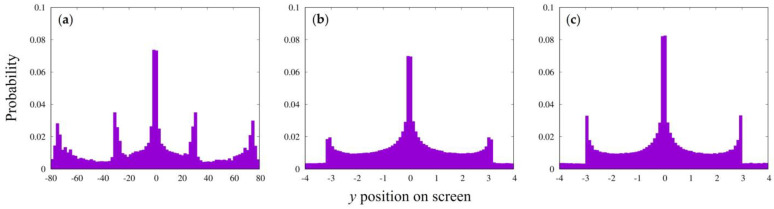
Interference pattern for other conditions with point emission particles. (**a**) Interference pattern with slit width of 1, slit spacing of 6, time step of 3, and interaction strength of 1.385, same as for Figure 3a. The emission position was *x_i_* = −30,000 and *con* = 10^−5^. Total numbers of emitted and arrived particles were 2,000,000 and 30,498, respectively. (**b**) Interference with slit width of 0.04, slit spacing of 0.06, emission position of 400, and degree of random motion *con* = 10^−5^. Other conditions were the same as those for Figure 3b. Total numbers of emitted and arrived particles were 2,000,000 and 379,524. (**c**) Interference with slit width of 0.04, slit spacing of 0.06, emission position of 300, and *con* = 10^−5^. Other conditions were the same as those for Figure 3c. Total numbers of emitted and arrived particles were 2,000,000 and 313,032, respectively.

Figure 5 shows examples of the interference patterns for the other slit conditions, which are quite similar to the patterns obtained for the parallel emission beams in Figure 3.

## 4. Discussion

The analogy of quantum-like behaviors in the biological and macroscopic world with quantum mechanics was discussed in many papers (see, e.g., [2,15]). By applying adaptive dynamics, we found that the quantum-like behavior came from the interactions with the environment [3,30] and proposed the following view: 

Everything is under the interaction network, with its environment producing the entanglement and its quantum-like behavior. As described in Section 1, the adaptive dynamical view enabled us to model and simulate such quantum-like behavior on the basis of classical dynamics. Thus, we came back to physics, asking whether this concept is applicable to a genuine quantum mechanical phenomenon, a two-slit interference, which was qualitatively successful, as shown in Section 3.

This successful demonstration using a particle model interacting with its environment does not necessarily prove that the quantum mechanics is based on such interaction network of particles with its environment, but still, this demonstration gives rise to the possibility that this can be the case. If we admit this possibility, we do not have to be annoyed by the strange property of duality, “particle and wave at the same time”, because adaptive dynamics gives observation dependence. In addition, by considering the stochastic nature of the adaptive dynamics, the apparent contradiction between the special relativity theory and quantum mechanics, i.e., the speed of light vs. instantaneous information transfer (see [31] for discussion), would be avoided.

Many researchers such as Nelson [29] and Nagasawa [32] have proposed stochastic equations equivalent to the Schrodinger equation to describe the particle behavior within the quantum mechanical framework. Their summing up of the stochastic equations for the two pathways may correspond to our assumption of a particle interacting with its environment, including the universe, which may produce the random walk behavior of a particle through the digitized interactions with all of the composite particles in our universe in addition to those in two-slit environments.

A specific slit condition with a specific time step and interaction strength was essential for the successful demonstration of the interference pattern. This coincides well with the adaptive dynamical nature of the two-slit problem; an interaction with the environment is essential. Correspondingly, a slight displacement of the particle emission position dramatically changed the resulting pattern (Figure 2d and Figure 4d). The actual experiments with the light wave show no such effect. We think that our simulation condition, especially the mode of interaction, has not yet fully represented the actual behavior of light. Such parameters for simulation conditions including the interaction potential and mode are imaginary at present, and further experimental investigation is necessary to determine if our adaptive dynamical view actually works as the principle of quantum mechanics.

A trial would be interesting to demonstrate the interference of two slits in a macroscopic experimental condition by referring to the two-slit situation considered in this study. Such macroscopic experimental set-up would be useful to understand and investigate the quantum-like behavior, or entanglement, especially for young researchers and students. Furthermore, our successful simulation examples provide them with useful tools to learn and examine the quantum-like behavior of the two-slit situation and other quantum mechanical phenomena. We have shown just a few successful patterns under limited conditions, which can be the start toward further searches of more realistic simulations.

Our simulation results are different from the experimental interference patterns in several points such as one-slit patterns (Figure 2b and Figure 4b) and the above-discussed slight displacement problem. We did not investigate them in detail. Moreover, our proposal based on adaptive dynamics apparently disagrees with many reports on the two-slit problem, showing the duality, especially considering the “which-path” experiments [28]. The corresponding simulation results (Figure 2c and Figure 4c) indicate its adaptive dynamical nature, which is different from the classical quantum mechanics. This means, on one hand, that our simulation set-up does not represent the quantum mechanical situation, but on the other hand, those experimental conditions in the “which-path” experiments should be reexamined carefully in view of the adaptive dynamics because the two conditions for the two-slit and “which-path” experiments are necessarily different even though the difference may be very minute.

In this respect, further refinements and investigations of the simulation conditions are necessary to propose adaptive dynamics as a probable candidate for quantum mechanics in addition to make the simulation a suitable educational tool of quantum mechanics.

## Data Availability

The data presented in this study are available from the corresponding authors upon reasonable request.
